# Survival Analysis in Gastrointestinal Neuroendocrine Carcinoma With Bone Metastasis at Diagnosis

**DOI:** 10.3389/fsurg.2022.820725

**Published:** 2022-01-28

**Authors:** Genlian Chen, Qiang Xu, Shengjun Qian, Zhan Wang, Shicheng Wang

**Affiliations:** ^1^Department of Orthopedics, People's Hospital of Pan'an County, Jinhua, China; ^2^Department of Orthopedics, Xuzhou Central Hospital, Xuzhou, China; ^3^Department of Orthopedic Surgery, The Second Affiliated Hospital, Zhejiang University School of Medicine, Hangzhou, China; ^4^Department of Orthopedic Surgery, Ningbo No.6 Hospital, Ningbo, China

**Keywords:** gastrointestinal, neuroendocrine carcinoma, bone metastasis, survival, risk factors

## Abstract

**Purpose:**

Gastrointestinal neuroendocrine carcinoma (NEC) with bone metastasis is rarely reported. The purpose of this study is to explore the prognosis and risk factors of such patients.

**Patients and Methods:**

We retrospectively reviewed patients diagnosed as gastrointestinal NECs with bone metastasis at diagnosis from 2010 to 2016 by using the Surveillance, Epidemiology and End Results (SEER) database. Predictors of overall survival (OS) and cancer-specific survival (CSS) were analyzed by univariable and multivariable Cox analyses. Kaplan–Meier plots were constructed to show the correlation between independent predictors and survival.

**Results:**

A total of 330 gastrointestinal NEC patients with bone metastasis at diagnosis were included for analysis. Over half of patients were male and older than 60 years old. The most common primary site of gastrointestinal NEC with bone metastasis was the pancreas. The prognosis of gastrointestinal NEC with bone metastasis (3-year OS and CSS rates: 16.7 and 17.0%) was very poor. On Cox multivariable analysis, age over 60 years old, no surgery, and lung metastasis were independent predictors of decreased OS and CSS.

**Conclusions:**

We identified three independent factors associated with prognosis among gastrointestinal NEC patients with bone metastasis, namely age, surgery, and lung metastasis. For younger gastrointestinal NEC patients with bone metastasis, surgical resection of primary tumors as well as actively treating lung metastasis might be useful for prolonging survival.

## Introduction

Neuroendocrine carcinoma (NEC) is a poorly differentiated subtype of neuroendocrine neoplasms (NENs) according to the 2019 World Health Organization (WHO) classification (1). Although NECs are commonly considered as rare, they have a rising incidence in recent years (2–4). NECs are characterized by poorly differentiated morphology as well as a high proliferation capacity (Ki−67 proliferation index > 20%) (5). Moreover, NECs are often presented with metastasis at diagnosis or advanced status and have a poor prognosis (6–8). Lung is the common site of NECs, while gastrointestinal NECs are rarely seen and account for about 35–55% of all extra-pulmonary NECs (9). Gastrointestinal NECs mainly arise from the esophagus, stomach, pancreas, colon and rectum and common metastatic sites for NECs include liver, lung and bone (8, 10).

Various treatment options are available for curative and palliative treatment of gastrointestinal NECs, including surgical resection, chemotherapy, and radiotherapy (11). However, treatment outcomes of gastrointestinal NECs remain poor (12). Due to the rarity of gastrointestinal NECs, there are limited researches regarding the treatment and survival of metastatic gastrointestinal NECs. As far as we know, clinical studies on survival analysis of gastrointestinal NECs with bone metastasis are lacking. Therefore, we conducted a population-based study by applying the Surveillance, Epidemiology and End Results (SEER) database to investigate the clinical characteristics and prognostic factors for gastrointestinal NECs with bone metastasis. Our findings may bring insight into gastrointestinal NECs with bone metastasis and provide a survival benefit on survival.

## Materials and Methods

### Study Population

This study retrospectively reviewed patients diagnosed as gastrointestinal NECs with bone metastasis at presentation by using the U.S National Cancer Institute's SEER database (Version 8.3.8) between 2010 and 2016. This population-based database collects information on cancer patients in 18 registries, covering approximately 30% of cancer cases in the U.S (www.seer.cancer.gov). Ethical review is not required for the present study because the public database does not contain information to identify patients.

Histological types were defined by the following International Classification of Diseases for Oncology, 3rd edition (ICD-O-3) code 8246/3 (neuroendocrine carcinoma, NOS). The primary sites were defined, including the esophagus, stomach, liver, pancreas, small intestine, cecum, appendix, colon, rectum, anus, gallbladder, biliary tract and other GI sites. Cases diagnosed not by histopathological examinations were excluded. In the present study, clinicopathological data, sociological data and treatment data were included for analysis. Surgery or radiotherapy in this study refers to the primary sites (13). Based on previous literature (14, 15), overall survival (OS) and cancer specific survival (CSS) are defined as the time from initial diagnosis to death due to any cause and NEC, respectively.

### Statistical Methods

All statistical analyses were performed by using IBM SPSS Statistics (Version 22). First, univariable Cox regression analyses were performed to select significant survival predictors. Then, significant risk factors from univariable analyses were integrated into multivariate Cox regression analysis to identify independent risk factors. Meanwhile, hazard ratios (HRs) and 95% confidence intervals (CIs) were calculated. Survival curves of independent risk factors were constructed with the Kaplan-Meier method. Variables with two-tailed *p* < 0.05 were considered statistically significant.

## Results

### Patients Characteristics

In total, 330 patients with gastrointestinal NEC and bone metastasis at diagnosis extracted from the SEER database were eligible for the analysis. Table 1 summarized the demographics of all patients. Of all patients, 79.1% were white and 62.7% were males. Over half of patients (62.1%) were aged over 60 years old. 25.2% (*n* = 83) of patients were located in the colon and rectum, 49.7% (*n* = 164) of patients were located in the pancreas, liver, stomach, and 18.31% of patients (*n* = 63) were located in other sites. Tumor size distribution was <5 cm 33.3%, ≥5 cm 25.8%, and unknown 40.9%. In terms of treatment methods, 34 (10.3%) patients received surgery, 108 (32.7%) received radiotherapy, and 180 (54.5%) received chemotherapy. Liver metastasis (76.1%) was more common than brain metastasis (6.4%) or lung metastasis (23.6%). Over half of the patients (55.5%) were married. One-year OS and CSS rate for all patients were 39.5 and 40%, respectively.

**Table 1:** Demographics of 330 gastrointestinal neuroendocrine carcinoma with bone metastasis at diagnosis.

**Variable**	**Value**
**Race**	
White	261 (79.1%)
Black	43 (13.0%)
Others	26 (7.9%)
**Gender**	
Female	123 (37.3%)
Male	207 (62.7%)
**Age (years)**	
≤60	125 (37.9%)
>60	205 (62.1%)
**Tumor location**	
Colon and rectum	83 (25.2%)
Pancreas, liver, stomach	164 (49.7%)
Others	83 (25.2%)
**Tumor size (cm)**	
<5	110 (33.3%)
≥5	85 (25.8%)
Unknown	135 (40.9%)
**Surgery**	
Yes	34 (10.3%)
No	296 (89.7%)
**Radiotherapy**	
Yes	108 (32.7%)
No	222 (67.3%)
**Chemotherapy**	
Yes	180 (54.5%)
No	150 (45.5%)
**Brain metastasis**	
No	294 (89.1%)
Yes	21 (6.4%)
Unknown	15 (4.5%)
**Liver metastasis**	
No	73 (22.1%)
Yes	251 (76.1%)
Unknown	6 (1.8%)
**Lung metastasis**	
No	234 (70.9%)
Yes	78 (23.6%)
Unknown	18 (5.5%)
**Marital status**	
Married	183 (55.5%)
Others	131 (39.7%)
Unknown	16 (4.8%)
**Dead**	
Yes	257 (77.9%)
No	73 (22.1%)
**1-year OS rate**	39.50%
**1-year CSS rate**	40.00%
**3-year OS rate**	16.70%
**3-year CSS rate**	17.00%

*OS, overall survival; CSS, cancer-specific survival*.

### Survival Analysis

On univariable analysis, variables found to be significantly associated with both OS and CSS were age, surgery, and lung metastasis (Table 2). There was no significant difference in OS or CSS in terms of race, gender, tumor location, tumor size, radiotherapy, chemotherapy, brain metastasis, liver metastasis, and marital status (Table 2).

**Table 2:** Univariate Cox analysis of survival in gastrointestinal neuroendocrine carcinoma with bone metastasis.

**Variable**	**OS**		**CSS**	
	**HR (95% CI)**	***P***	**HR (95% CI)**	***P***
**Race**				
White	1		1	
Black	1.139 (0.792–1.639)	0.482	1.102 (0.754–1.611)	0.615
Others	0.921 (0.575–1.475)	0.732	0.930 (0.580–1.490)	0.762
**Gender**				
Female	1		1	
Male	0.961 (0.745–1.239)	0.758	0.984 (0.758–1.277)	0.902
**Age (years)**				
≤60	1		1	
>60	1.355 (1.050–1.749)	0.2	1.317 (1.016–1.708)	0.037
**Tumor location**				
Colon and rectum	1		1	
Pancreas, liver, stomach	0.801 (0.596–1.076)	0.14	0.801 (0.594–1.080)	0.146
Others	0.830 (0.586–1.174)	0.292	0.817 (0.572–1.167)	0.265
**Tumor size (cm)**				
<5	1		1	
≥5	1.335 (0.969–1.840)	0.077	1.303 (0.936–1.814)	0.117
**Surgery**				
Yes	1		1	
No	1.811 (1.168–2.808)	0.008	1.781 (1.148–2.765)	0.01
**Radiotherapy**				
Yes	1		1	
No	1.000 (0.772–1.296)	0.998	0.992 (0.762–1.293)	0.955
**Chemotherapy**				
Yes	1		1	
No	0.982 (0.763–1.263)	0.887	0.942 (0.727–1.221)	0.651
**Brain metastasis**				
No	1		1	
Yes	1.412 (0.883–2.257)	0.15	1.585 (0.977–2.570)	0.062
**Liver metastasis**				
No	1		1	
Yes	1.136 (0.842–1.532)	0.406	1.145 (0.842–1.556)	0.389
**Lung metastasis**				
No	1		1	
Yes	1.519 (1.145–2.015)	0.004	1.560 (1.167–2.086)	0.003
**Marital status**				
Married	1		1	
Others	1.020 (0.792–1.314)	0.878	0.992 (0.766–1.286)	0.953

**Figure 1:**
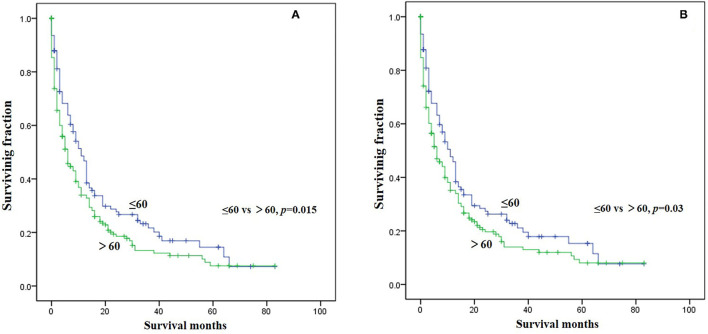
Kaplan-Meier method estimated survival in gastrointestinal NEC with bone metastasis at diagnosis stratified by age. **(A)** OS, **(B)** CSS (OS, overall survival; CSS, cancer-specific survival).

**Figure 2:**
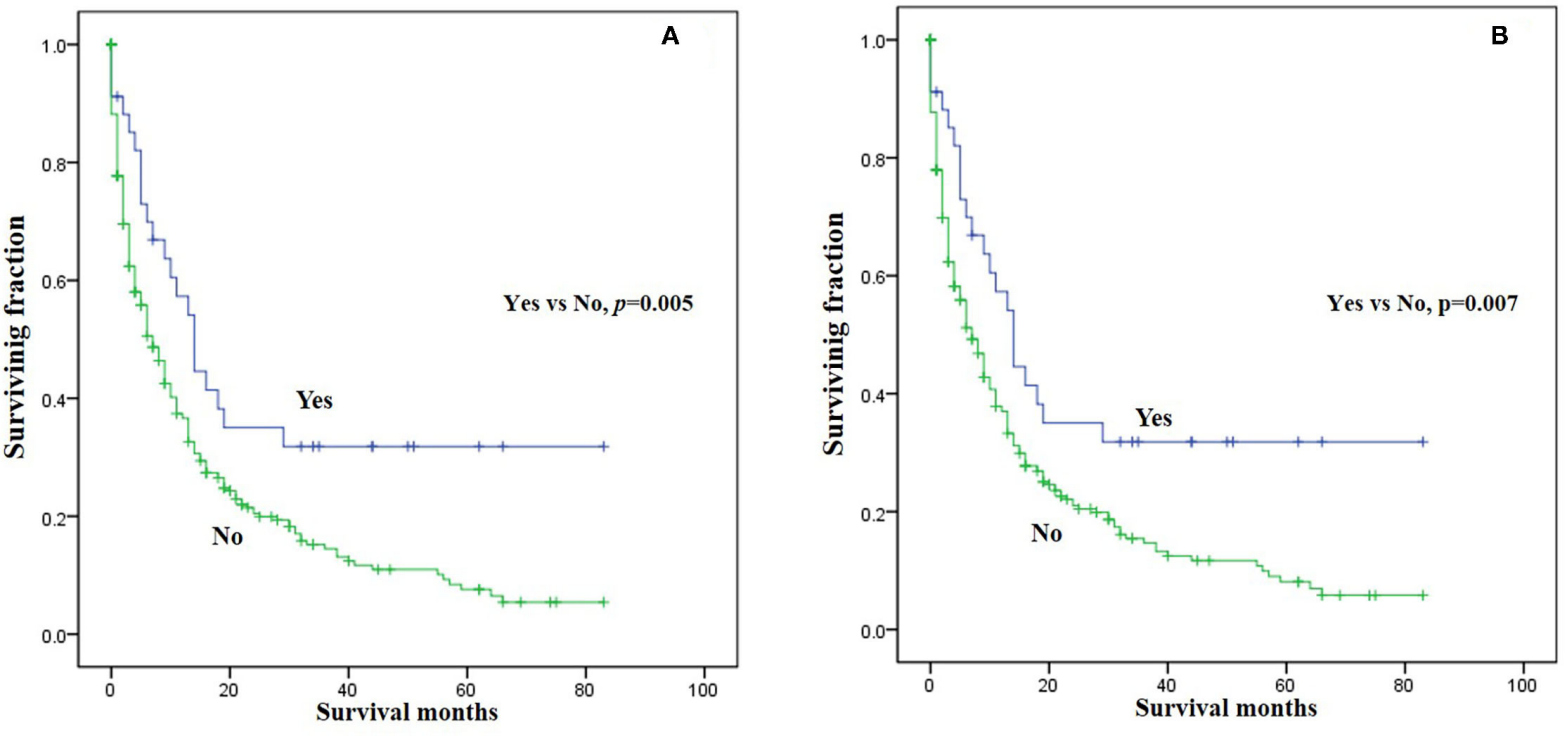
Kaplan-Meier method estimated survival in gastrointestinal NEC with bone metastasis at diagnosis stratified by surgery. **(A)** OS, **(B)** CSS (OS, overall survival; CSS, cancer-specific survival).

**Figure 3:**
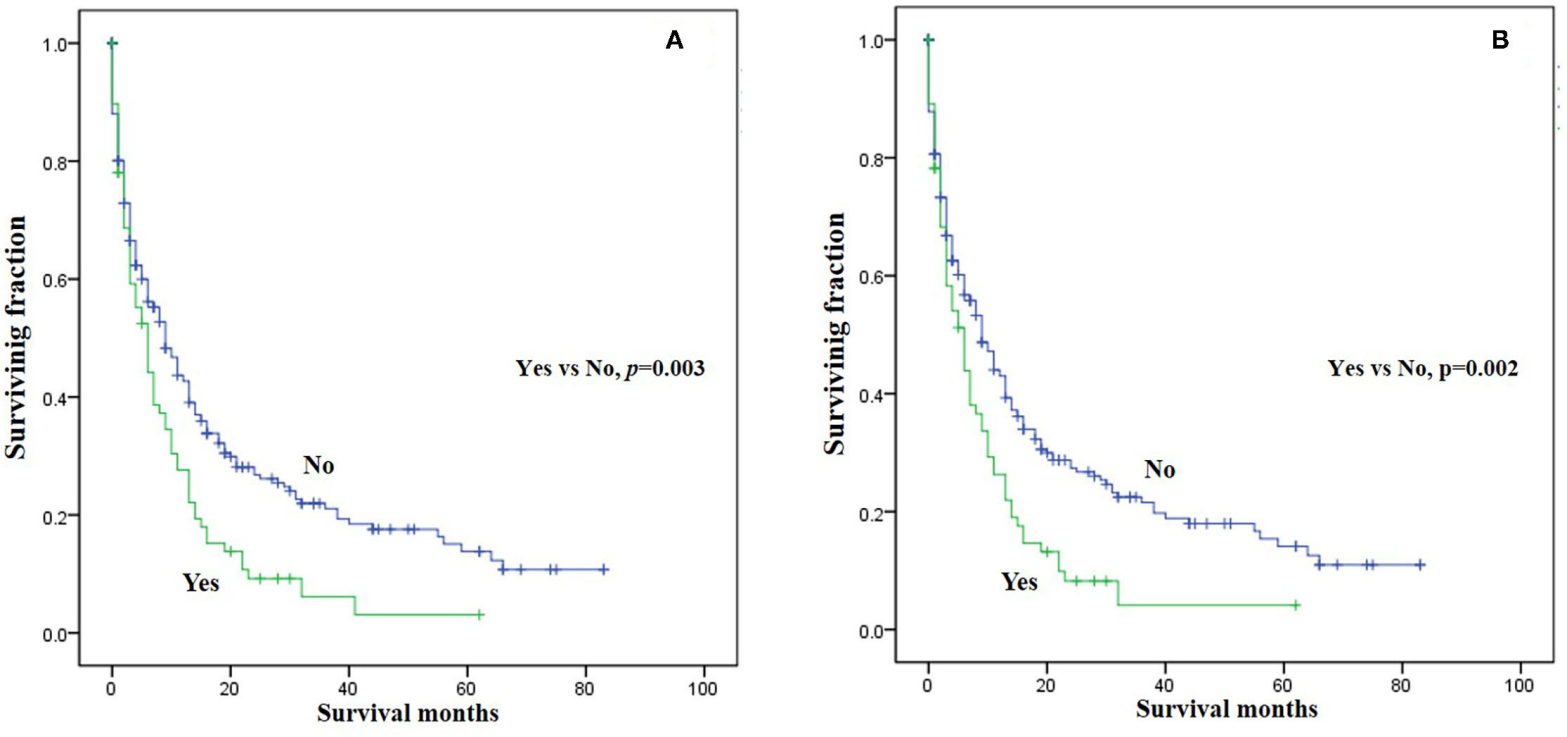
Kaplan-Meier method estimated survival in gastrointestinal NEC with bone metastasis at diagnosis stratified by lung metastasis. **(A)** OS, **(B)** CSS (OS, overall survival; CSS, cancer-specific survival).

Three significant risk factors for OS and CSS identified in the univariable Cox analysis were integrated into the multivariable analysis. Multivariable analysis revealed age, surgery, and lung metastasis were significant predictors for both OS and CSS (Table 3). Additionally, the Kaplan–Meier survival curves showed that patients with age ≤60 years old (Figure 1), surgery (Figure 2), or no lung metastasis (Figure 3), had increased OS and CSS.

**Table 3:** Multivariate Cox regression analysis of survival in gastrointestinal neuroendocrine carcinoma with bone metastasis.

**Variable**	**OS**		**CSS**	
	**HR (95% CI)**	***P***	**HR (95% CI)**	***P***
**Age (years)**				
≤60	1		1	
>60	1.376 (1.064–1.779)	0.015	1.328 (1.024–1.723)	0.033
**Surgery**				
Yes	1		1	
No	1.738 (1.120–2.698)	0.014	1.726 (1.111–2.681)	0.015
**Lung metastasis**				
No	1		1	
Yes	1.534 (1.155–2.038)	0.003	1.564 (1.169–2.092)	0.003

## Discussion

Previous studies indicated that NEC patients were prone to metastasis. Although some studies reported the prognosis of gastrointestinal NEC, few studies have been conducted on the prognosis of gastrointestinal NEC with bone metastasis. To our knowledge, this is the largest study investigating the clinical characteristics, and prognosis of gastrointestinal NEC patients with bone metastasis. The 3-year CSS rate of these patients was only 17%, which suggested a poor outcome. Moreover, significant independent predictors affecting gastrointestinal NEC with bone metastasis included age, lung metastasis, and surgery. The results of this study may help clinicians develop appropriate treatment strategies and provide reasonable treatment suggestions for patients.

No significant difference on survival was observed in univariate analysis among patients with different races. Interestingly, some studies on lung neuroendocrine neoplasms revealed race was an independent survival predictor (16, 17). Patients over 60 years old are generally considered the elderly. Based on previous literature (18, 19), we divided the patients' age into >60 years old and ≤60 years old for convenient analysis. Multivariate analysis revealed that age under 60 years were associated with improved OS and CSS, which was in line with previous studies (20–22). There is no significant difference in survival time between male and female patients. The most common primary site of gastrointestinal NEC with bone metastasis was the pancreas. Additionally, no significant difference was observed in OS and CSS among patients with different primary tumor locations. Based on previous literature (23, 24), we divided the tumor size into ≥5 cm and <5 cm for convenient analysis. For many primary malignant tumors, tumor size is often one of the important factors affecting the prognosis of patients (25, 26). In contrast to our traditional knowledge, we noted that tumor size in the current study was not correlated with survival. Further studies are needed to confirm this finding.

Treatment patterns and outcomes of gastrointestinal NEC patients especially metastatic ones have not been well-described. Our multivariable results highlighted the role of surgical resection in prolonging the survival, which was consistent with the situation of non-metastatic patients (12). Sorbye et al. (9) recommended that chemotherapy treatment should be performed for advanced gastrointestinal NEC patients. However, our studies found that chemotherapy or radiotherapy did not improve the prognosis of those patients. Although liver metastasis was most common in gastrointestinal NEC patients with bone metastasis, it was not an independent risk factor for prognosis. Additionally, the presence of brain metastasis was not associated with a decreased survival. Of note, these patients were secondly complicated with lung metastasis, which was recognized as an independent risk factor for the decreased prognosis. Therefore, actively treating lung metastasis may have positive effect on survival. Marital status had no association with survival in this study. In accordance with these findings, we suggested that for younger gastrointestinal NEC patients with bone metastasis, surgical resection of primary tumors as well as actively treating lung metastasis might be useful for prolonging survival.

Some limitations of the present study should be noted. First, this study has retrospective nature, which can lead to bias. Second, detailed therapy information is not available in the database. Third, local recurrence or distal metastasis data during follow-up were not recorded in the database. Additionally, due to the small sample size and few independent risk factors in this study, we did not build a prediction model. Future studies can attempt to build predictive models. Although this study has these limitations, it has important implications for managing these patients.

## Conclusions

This is the largest study of survival analysis on gastrointestinal NEC patients with bone metastasis. Age, surgery and lung metastasis were identified as independent risk factors of survival. Resection of primary tumors as well as actively treating lung metastasis can prolong the survival of this special cohort. However, multicenter co-operative studies should be performed to confirm our findings in the future.

## Data Availability Statement

The raw data supporting the conclusions of this article will be made available by the authors, without undue reservation.

## Ethics Statement

Ethical review and approval was not required for the study on human participants in accordance with the local legislation and institutional requirements. Written informed consent for participation was not required for this study in accordance with the national legislation and the institutional requirements.

## Author Contributions

SW and ZW conceived and designed the study. GC, QX, and SQ collected the data. GC, QX, and SQ performed the statistical analysis. GC wrote the manuscript and SW and ZW revised it. All authors read and approved the final manuscript.

## Funding

This work was supported by the China Postdoctoral Science Foundation (2021M692792), National Natural Science Foundation of China (82103499), and Zhejiang Provincial Natural Science Foundation (LQ22H160040).

## Conflict of Interest

The authors declare that the research was conducted in the absence of any commercial or financial relationships that could be construed as a potential conflict of interest.

## Publisher's Note

All claims expressed in this article are solely those of the authors and do not necessarily represent those of their affiliated organizations, or those of the publisher, the editors and the reviewers. Any product that may be evaluated in this article, or claim that may be made by its manufacturer, is not guaranteed or endorsed by the publisher.
